# Executive function performance in Chinese youth ice hockey players: a comparison between expert and novice groups

**DOI:** 10.3389/fpsyg.2026.1670150

**Published:** 2026-02-03

**Authors:** Jin Wang, Xiaolei Yang, Peng Shi

**Affiliations:** 1 School of Leisure Sports, Chengdu Sport University, Chengdu, China; 2 School of Physical Education, Liaoning Normal University, Dalian, China; 3 School of Physical Education, Shandong University of Technology, Zibo, China

**Keywords:** executive function, ice hockey player, inhibition, shifting, updating, youth

## Abstract

**Objective:**

This study aims to explore the executive function advantages of Chinese youth ice hockey players and provide a theoretical basis for the selection and training of ice hockey players.

**Methods:**

A total of 132 youth ice hockey players were recruited and divided into an expert group (65 players, mean age 15.8 years, mean training duration 6.7 years) and a novice group (67 players, mean age 16.2 years, mean training duration 3.2 years). The Flanker task, 2-back task, and More-odd shifting task were used to measure inhibition, updating, and shifting functions, respectively. Statistical analyses were performed using the Mann–Whitney *U* test, independent-samples *t*-test and Pearson correlation analysis.

**Results:**

Athletes exhibit a speed-accuracy trade-off. The expert group showed significantly shorter response times than the novice group in the congruent condition (*Z* = −2.681, *p* = 0.007) and incongruent condition (*Z* = −1.998, *p* = 0.046) of the Flanker task, the 2-back task (*Z* = −2.378, *p* = 0.017), and the size-parity shifting condition of the More-odd shifting task (*Z* = −2.548, *p* = 0.011). However, there were no significant differences in accuracy between the two groups across all tasks. In addition, training duration was significantly negatively correlated with response times in executive function tasks (inhibition congruent condition: *r* = −0.450; inhibition incongruent condition: *r* = −0.267; updating task: *r* = −0.257; shifting task: *r* = −0.185; all *p* < 0.05).

**Conclusion:**

Youth ice hockey players in the expert group demonstrate more superior executive functions, and there is a significant negative correlation between training duration and response time in executive function tasks.

## 1 Introduction

On the ice hockey rink, top players can always capture the dynamics of the game in an instant, flexibly allocate their focus, and resist external interference, thereby quickly formulating or adjusting tactics (Goodreau, 2012). This highly praised sports IQ in the field of sports actually corresponds to the concept of brain executive function in the realm of neuropsychology (Kilger and Blomberg, 2020). In addition, the brain’s executive function is closely associated with athletes’ information search and processing, attention allocation, and motor decision-making, and athletes with higher levels of executive function tend to possess more excellent motor decision-making capabilities (Cao et al., 2025; Heisler et al., 2023; Vestberg et al., 2017). Therefore, researchers have generally focused on athletes’ executive functions, aiming to confirm the advantages in executive functions among elite athletes and further propose training strategies based on executive functions, so as to enhance the pertinence of players’ training.

The so-called executive function is a high-level cognitive function of humans, generally referring to the cognitive ability of individuals to control the collaborative operation of multiple cognitive processing processes in a flexible and optimized manner when achieving a specific goal (Anderson, 2010). Simply put, executive function is the advanced control and coordination function of the human brain (Friedman and Robbins, 2022). Executive function consists of three dimensions: inhibition, updating, and shifting, which are both related and independent of each other, each playing an important role in complex executive tasks (Baggetta and Alexander, 2016). The inhibition refers to an individual’s ability to actively suppress and control their own automatic, dominant, or irrelevant cognitive responses and behavioral tendencies. Its core function is to eliminate interference, thereby ensuring the smooth execution of goal-oriented behaviors. The updating refers to the process of continuously modifying the contents of working memory based on newly presented information, reflecting people’s ability to constantly revise the contents in memory. The shifting refers to the control mechanism of endogenous attention guided by instructions, that is, the process of switching between two tasks when they compete for the same cognitive resources.

Currently, the results of relevant meta-analyses (Kalén et al., 2021; Logan et al., 2023; Scharfen and Memmert, 2019) indicate that experts and elite athletes possess more superior cognitive functions with small to moderate effect sizes, particularly in terms of attention control and cognitive flexibility. In addition, Ren et al. (2025) further explored whether athletes possess superior executive functions. The results of a meta-analysis incorporating 41 studies revealed that athletes exhibit higher inhibition control and working memory, and such executive control performance increases with the accumulation of sports experience. In recent years, in ice hockey clubs in Europe and America, many researchers have conducted systematic and in-depth studies on the executive functions of the brain in elite athletes and non-elite athletes using cognitive neuroscience tests. For example, the study by Tamás et al. (2024) found that near-elite ice hockey players have higher cognitive regulation skills. Another example is that Lundgren et al. (2016) used the D-KEFS test system to assess the executive functions of 48 ice hockey players, and found that ice hockey players performed more excellently in design fluency. However, the performance of elite players was not significantly lower than that of players in lower-level leagues.

Based on existing literature, the following limitations can be identified in current research: Firstly, there is a relative scarcity of studies on the executive functions of ice hockey players, with a particular lack of evidence from Chinese samples. Secondly, the tools currently used to assess the executive functions of ice hockey players are not the commonly adopted computer-based testing tasks such as Flanker, 2-back, and More-shifting. As a result, it is impossible to determine which dimension of executive function ice hockey players excel in.

Based on this, this study tests the executive functions of Chinese adolescent ice hockey players, aiming to reveal the advantages of executive functions in adolescent ice hockey players, explore the specific dimensions of executive functions where ice hockey players perform more excellently, and provide theoretical and practical guidance for the selection and training of ice hockey players.

## 2 Methods

### 2.1 Study design

This study adopted a cross-sectional research design to investigate the differences in executive functions between elite and novice adolescent ice hockey players, and further explore the correlation between training duration and executive function. Firstly, information such as the athletes’ sports level, training years, weekly training frequency, and single training duration was collected and statistically organized. Secondly, the included athletes were divided into an elite group and a novice group based on the above-mentioned kinematic information. Thirdly, the Flanker task, 2-back task, and More-odd Shifting task were used to measure the three dimensions of executive functions, namely inhibition, updating, and shifting, respectively. Finally, inter-group comparison and correlation analysis were adopted for data analysis.

### 2.2 Participants

This study used G*Power 3.1 software to calculate the sample size. Drawing on the meta-analysis results by Scharfen and Memmert (2019), the effect size (*ES* = 0.22) of the superior cognitive performance among experts and elite athletes was extracted. With *α* set at 0.05 and 1-*β* at 0.8, it was calculated that a total of 130 participants would be required. In addition, to ensure the sufficiency of the sample, a 10% possible inefficiency rate was set, so a total of 143 participants would be needed. A total of 143 adolescent ice hockey players were recruited from Shenyang, China, for this study. They were divided into an expert group and a novice group based on criteria such as sports rank, average years of training, weekly training frequency, and duration of each training session. Among them, the 70 players in the expert group were mainly from the Shenyang Municipal Team, all holding the title of National Level 1 Athletes. The 73 players in the novice group were mainly from two adolescent ice hockey clubs in Shenyang, without any sports rank. In addition, due to the special nature of ice hockey, there are very few female participants in ice hockey in China. Therefore, all participants included in this study were male players.

In this study, participants were screened according to the following inclusion criteria: (1) The response time, which effectively reflects cognitive function, should be > 150 ms (Tsai et al., 2014). A response time lower than this value was judged as an accidental key press or random key pressing; (2) Normal uncorrected or corrected visual acuity, without blurred vision, color blindness, or color weakness; (3) Stable mood during the computer task tests, without obvious negative emotions such as tension, impatience, or anxiety. Ultimately, out of 143 participants, 132 were selected for the statistical analysis of executive function task performance in the data analysis. These included 65 players in the expert group and 67 players in the novice group. The inclusion criteria of this study are mainly used for the preliminary screening of participants to ensure the validity and homogeneity of the sample data.

This study was conducted in strict accordance with the core guidelines of the Declaration of Helsinki. Before the official launch of the study, all participants were fully informed of key information such as the research purpose, procedures, potential risks and rights, and their voluntarily signed informed consent forms were obtained. Meanwhile, the research protocol has been strictly reviewed and approved by the Research Ethics Committee of Chengdu Sport University (Approval No.: 2025229). Throughout the implementation of the study, the protection of the legitimate rights and interests, physical and mental health, and personal privacy of the participants has always been given top priority to ensure the compliance and ethics of the research conduct.

### 2.3 Methods

#### 2.3.1 Data collection process

A standardized experimental procedure in sports psychology was adopted in this study for data collection, and this procedure has been applied in numerous relevant studies (Gao et al., 2025; Shi et al., 2023). One researcher sequentially leads the participants into the sports psychology laboratory. The laboratory environment is quiet with no strong light stimulation. Firstly, the participants are asked to fill out a basic information questionnaire, which includes details such as age, sports level, years of sports participation, weekly exercise frequency, and experience in participating in important competitions. Secondly, under the guidance of the researcher, the participants familiarize themselves with the task operation process, and the formal test can only be conducted after they have mastered the process. The order of the test tasks is Flanker, 2-back, and More-odd shifting. After completing one task, the participants take a 1-min break before proceeding to the next task. Finally, after the test, the Positive and Negative Affect Schedule (PANAS) is distributed to the participants to investigate their emotional state during the test, so as to check whether they have excessive negative emotions such as tension and anxiety during the executive function test.

#### 2.3.2 Variables and tools

##### 2.3.2.1 Executive function test procedure

In this study, to measure Participants’ inhibition, updating, and shifting functions, we adopted the Flanker task, 2-back task, and More-odd shifting task designed by Chen et al. (2014). These tasks were programmed using the psychological programming tool Psychopy, and after three rounds of optimization and adjustment, the corresponding test procedures were finally established. The Flanker task, 2-back task, and More-odd shifting task are psychological test tasks widely used to measure executive function, and they have been proven to have high reliability and validity (Ren et al., 2025).

###### 2.3.2.1.1 Flanker task

At the start of the test, a fixation point will be presented in the center of the screen for 500 ms, followed by a string of characters for 1,000 ms, with an inter-stimulus interval of 2,000 ms. The strings include two conditions: congruent (e.g., “FFFFF” or “LLLLL”) and incongruent (e.g., “LLFLL” or “FFLFF”). Participants are required to place their left and right index fingers on the “F” and “L” keys of the keyboard, respectively, and judge the middle letter of the string as quickly and accurately as possible. If the middle letter is “F,” press the “F” key with the left index finger; if it is “L,” press the “L” key with the right index finger. Before the formal test, the researcher will guide the participants through 12 practice trials to ensure familiarity with the task. The formal test consists of 32 judgment trials, with the two conditions randomized and presented with equal probability. Drawing on previous studies (Chen et al., 2014; Ma et al., 2022; Zhang et al., 2009), test performance will be measured by response time and accuracy under both congruent and incongruent conditions. Shorter RTs and higher accuracy rates indicate better inhibition control.

###### 2.3.2.1.2 2-back task

This test selects seven English letters, namely “Q, E, R, G, T, Y, W”, as the stimulus materials. Each letter is randomly presented individually in the center of the screen. The presentation duration of each stimulus letter is 1,000 ms, and the inter-stimulus interval is 2,000 ms. During the test, Participants are required to place their left and right index fingers on the “F” key and “J” key of the keyboard, respectively. In the process of the task, Participants need to carefully watch the presented letters. If the presented letter is the same as the second letter from the last one presented earlier, they should press the “F” key with their left index finger; if not, they should press the “J” key with their right index finger. Participants are required to make judgment responses as quickly as possible while ensuring accuracy. Before the formal test, 12 practice trials are conducted. The formal test includes 48 judgment responses. Referring to previous studies (Chen et al., 2014; Ma et al., 2022; Zhang et al., 2009), response time and accuracy are selected to indicate Participants’ test performance. Generally speaking, the shorter the response time and the higher the accuracy, the better the Participants’ performance in the working memory task.

###### 2.3.2.1.3 More-odd shifting task

In this test, numbers from 1 to 9 (excluding 5) are randomly presented one by one on the screen as stimuli. These numerical stimuli appear sequentially in the center of the screen with duration of 2,000 ms, and the interval between stimuli is 3,000 ms. Participants are required to place their left and right index fingers on the “F” and “J” keys of a standard keyboard respectively, and make the following three types of judgment responses to the stimulus numbers: First, numerical attribute judgment: when a black number is presented, a size judgment task is performed (press the “F” key if the number is less than 5, and press the “J” key if it is greater than 5); Second, parity attribute judgment: when a green number is presented, a parity judgment task is executed (press the “F” key for odd numbers and the “J” key for even numbers); Third, task switching judgment: the type of the current task is determined by the color clue. If a black number is presented, the numerical attribute judgment is carried out; if a green number is presented, the parity attribute judgment is conducted. Participants need to shorten the response time as much as possible on the basis of ensuring the accuracy of key presses. The test process consists of two stages: a pre-test and a formal test. The pre-test stage mainly involves 12 adaptive practices for each task type; the formal test stage includes 16 numerical judgment tasks, 16 parity attribute judgment tasks, and 32 size-parity mixed judgment tasks. Among them, the entire test includes 16 shifts. Referring to previous studies (Chen et al., 2014; Ma et al., 2022; Zhang et al., 2009), the test results are reflected by the response time and accuracy under the conditions of size judgment, parity judgment, and size-parity shift. Generally speaking, the shorter the response time and the higher the accuracy, the better the performance in the cognitive flexibility task.

##### 2.3.2.2 Emotional state during the executive function test

This study employed the PANAS developed by Watson et al. to measure the emotional states of participants after they completed the executive function test (Zhang, 2005). The scale, revised by Huang et al. (2003), has been applicable to the assessment of emotional states in the Chinese population. Among its psychometric properties, the Cronbach’s *α* coefficients for positive affect and negative affect are 0.85 and 0.83, respectively; the test–retest reliability r for both is 0.47. With the SCL-90 scale as the criterion, negative affect shows a moderate to high degree of correlation with it (*r* = 0.43 ~ 0.65), indicating that the scale has good reliability and validity. The PANAS consists of 20 words describing different feelings and emotions, including a positive affect factor and a negative affect factor, each comprising 10 adjectives. In this study, participants whose negative affect scores were higher than their positive affect scores were excluded to control for the negative impact of obvious negative emotions such as tension, irritability, and anxiety on executive function tests.

##### 2.3.2.3 Questionnaire on participants’ basic information

A basic information questionnaire for participants was developed in this study with reference to previous studies (Gao et al., 2025; Shi et al., 2023) to investigate the fundamental information of adolescent ice hockey players. The main contents of the questionnaire included the following aspects: (1) age and the club or sport team to which they belong; (2) sports rank, which was used to understand the participants’ ice hockey technical proficiency; (3) years of participation in ice hockey, reflecting the duration of their engagement in ice hockey; (4) weekly frequency of ice hockey training or competitions, embodying the regularity of their sports participation; (5) experience of participating in major ice hockey events, for grasping their past involvement in official competitions. In this study, with reference to previous studies (Shi et al., 2023, 2022), adolescent ice hockey players were divided into an expert group and a novice group based on the above-mentioned athlete information.

### 2.4 Statistical methods

In this study, SPSS 25.0 software was used for relevant data processing and statistical analysis. For continuous variables, mean (*M*) and standard deviation (SD) were used for descriptive statistics; for categorical variables, frequency and percentage were used for descriptive statistics. A one-sample Kolmogorov–Smirnov test combined with P-P plots and Q-Q plots was used to assess the normality of the data. The results showed that the data related to athletes’ sports information approximately conformed to a normal distribution, while the data pertaining to executive function did not follow a normal distribution. Therefore, this study adopted an independent-samples *t*-test to conduct inter-group comparative analysis of the sports information data between the expert group and the novice group, and used the Mann–Whitney *U* test to perform inter-group comparison of the executive function data between the two groups. In addition, the present study adopted *r* to represent the effect size for the between-group comparative analysis of executive function, with the calculation formula being *r* = |*Z*|/ $\sqrt{N}$ , where |*Z*| denotes the absolute value of the *Z*-value derived from the Mann–Whitney *U* test and *N* represents the total sample size of the two groups. The study referred to Cohen’s classification criteria, where an *r* value of 0.5 indicates a large effect, an *r* value of 0.3 indicates a moderate effect, and an *r* value of 0.1 indicates a small effect (Gignac and Szodorai, 2016). Finally, Pearson correlation analysis was used to explore the relationship between the ice hockey training duration of adolescent ice hockey players and their executive function task performance after controlling for relevant demographic factors. In addition, the present study adopted Pearson’s correlation analysis to explore the correlation between response time and accuracy of athletes’ executive functions. If the correlation between response time and accuracy was low (with *r* approaching 0), it indicated that the participants did not sacrifice accuracy for speed or speed for accuracy, i.e., there was no significant speed-accuracy trade-off. For missing values, the linear interpolation method was used for imputation. The significance level for all the above statistical methods was set at *α* = 0.05.

## 3 Results

### 3.1 Basic information of the participants

Based on indicators including sports rank, average training years, weekly training frequency, and single training duration, the participants were divided into an expert group and a novice group. Details regarding the basic information of the two groups and the results of the inter-group comparative analysis are presented in Table 1. Specifically, the athletes in the expert group were mainly recruited from the Shenyang Municipal Team, all holding the title of National First-Class Athlete. They underwent training six times a week, with each session lasting 3 h, and all had participated in national-level competitions and achieved favorable results. In contrast, the 73 athletes in the novice group were primarily from two youth ice hockey clubs in Shenyang, without any official sports rank. They trained three times a week, with each training session lasting 1.5 h. Given that both the expert group and the novice group received unified training protocols, their weekly training frequency and single training duration were consistent within each respective group. In addition, the average age of the expert group was (15.29 ± 1.27) years, while that of the novice group was (15.54 ± 1.01) years, with no significant difference observed between the two groups (*t* = −1.230, *p* = 0.221). The average training years of the expert group stood at (5.22 ± 0.99) years, compared with (4.66 ± 1.62) years of the novice group, indicating that the expert group had a significantly longer average training duration than the novice group (*t* = 2.381, *p* = 0.019).

**Table 1:** Basic information of the expert group and the novice group.

Variables	Expert group	Novice group	*t*	*p*
Age	15.29 ± 1.27	15.54 ± 1.01	−1.230	0.221
Average training years	5.22 ± 0.99	4.66 ± 1.62	2.381	0.019
Weekly training frequency	6.00 ± 0.00	3.00 ± 0.00	—	—
Single training duration	3.00 ± 0.00	1.60 ± 0.00	—	—

### 3.2 Speed-accuracy trade-off in executive function

The results of the correlation analysis on response time and accuracy of executive function showed that there was a significant positive correlation between response time and accuracy under the congruent condition of inhibition control (*r* = 0.237, *p* < 0.01). A significant positive correlation was also observed between response time and accuracy in working memory (*r* = 0.184, *p* < 0.05). Additionally, response time and accuracy under the Parity shifting condition of cognitive flexibility were significantly positively correlated (*r* = 0.355, *p* < 0.01). In summary, athletes exhibited a speed-accuracy trade-off in the executive function test tasks. The results of the study are detailed in Table 2.

**Table 2:** Correlation analysis results of executive function response time and accuracy.

Variables	Congruent ACC	Incongruent ACC	Working memory ACC	Size shifting ACC	Parity shifting ACC	Size and parity shifting ACC
Congruent RT	0.237**					
Incongruent RT		−0.002				
Working memory RT			0.184*			
Size shifting RT				0.165		
Parity shifting RT					0.355**	
Size and parity shifting RT						0.060

RT, response time; ACC, accuracy; *indicates *p* < 0.05; **indicates *p* < 0.01.

### 3.3 Intergroup comparative analysis of inhibition function between expert group and novice group

In terms of the Flanker task performance of ice hockey players, both the expert group with rich experience and the novice group new to the field showed similar patterns: under the incongruent condition, not only was their reaction speed slower than that under the congruent condition, but their accuracy was also lower. This indicates that both groups of athletes have significant inhibition effects (Table 3). A further comparison of the specific data between the two groups (Table 3) reveals that in terms of response time, the expert group performed significantly better than the novice group—the expert group reacted more quickly both under the congruent condition (*Z* = −2.681, *p* = 0.007) and the incongruent condition (*Z* = −1.998, *p* = 0.046). The effect size reached a small-to-medium level (*r* = 0.233 for congruent RT and *r* = 0.174 for incongruent RT). However, in terms of accuracy, there was no statistically significant difference between the two groups. The performance of the expert group and the novice group was relatively close both under the congruent condition (*Z* = −0.044, *p* = 0.965) and the incongruent condition (*Z* = −0.152, *p* = 0.879).

**Table 3:** Results of intergroup comparative analysis on the Flanker task between expert group and novice group.

Variables	EG		NG		*Z*	*p*	*ES r*
	*Mdn (Q1, Q3)*	Rank mean	*Mdn (Q1, Q3)*	Rank mean			
Congruent RT	610.25 (546.69, 694.97)	57.04	647.25 (585.53, 753.02)	74.83	−2.681	0.007	0.233
Congruent ACC	93.75 (87.50, 100.00)	65.86	93.75 (87.50, 100.00)	66.14	−0.044	0.965	0.004
Incongruent RT	640.75 (566.88, 696.87)	59.32	674.78 (599.77, 766.56)	72.58	−1.998	0.046	0.174
Incongruent ACC	93.75 (84.38, 100.00)	65.51	93.75 (85.94, 100.00)	66.48	−0.152	0.879	0.013

EG, expert group; NG, novice group; M, mean; SD, standard deviation; RT, response time; ACC, accuracy; Mdn, median; *Q1*, first quartile; *Q3*, third quartile; *ES r* = effect size.

### 3.4 Intergroup comparative analysis of updating function between expert group and novice group

The results of the intergroup comparative analysis of the 2-back task between the expert group and the novice group (Table 4) showed that the response time of the expert group in the 2-back task was significantly shorter than that of the novice group (*Z* = −2.378, *p* = 0.017). The effect size reached a small-to-medium level (*r* = 0.207). However, there was no significant difference in accuracy between the two groups (*Z* = −0.164, *p* = 0.870).

**Table 4:** Results of intergroup comparative analysis on the 2-back task between expert group and novice group.

Variables	EG		NG		*Z*	*p*	*ES r*
	*Mdn (Q1, Q3)*	Rank mean	*Mdn (Q1, Q3)*	Rank mean			
RT	576.64 (312.85, 768.10)	58.46	628.50 (500.54, 914.59)	74.30	−2.378	0.017	0.207
ACC	54.35 (47.83, 61.96)	65.95	54.38 (47.83, 67.39)	67.04	−0.164	0.870	0.014

EG, expert group; NG, novice group; M, mean; SD, standard deviation; RT, response time; ACC, accuracy; Mdn, median; *Q1*, first quartile; *Q3*, third quartile; *ES r*, effect size.

### 3.5 Intergroup comparative analysis of shifting function between expert group and novice group

Whether it is the expert group or the novice group, the response time of athletes in size and parity shifting is significantly longer than that in size shifting and parity shifting alone. Meanwhile, the accuracy rate in size and parity shifting is significantly lower than that in the latter two single shifting tasks (Table 5). The results of the inter-group comparative analysis on the performance of the expert group and the novice group in the More-odd shifting task (Table 5) indicate that the response time of the expert group in size and parity shifting is significantly shorter than that of the novice group (*Z* = −2.548, *p* = 0.011). The effect size reached a small-to-moderate level (*r* = 0.222). Nevertheless, there are no significant differences between the two groups in terms of the response time of size shifting (*Z* = −0.803, *p* = 0.422), the accuracy rate of size shifting (*Z* = −0.444, *p* = 0.657), the response time of parity shifting (*Z* = −0.952, *p* = 0.341), the accuracy rate of parity shifting (*Z* = −0.329, *p* = 0.743), and the accuracy rate of size and parity shifting (*Z* = −0.826, *p* = 0.409).

**Table 5:** Results of intergroup comparative analysis on the more-odd shifting task between expert group and novice group.

Variables	EG		NG		*Z*	*p*	*ES r*
	*Mdn (Q1, Q3)*	Rank mean	*Mdn (Q1, Q3)*	Rank mean			
Size shifting RT	698.81 (630.00, 781.63)	61.83	724.38 (654.60, 779.32)	67.09	−0.803	0.422	0.070
Size shifting ACC	93.75 (87.50, 100.00)	63.09	93.75 (87.50, 100.00)	65.87	−0.444	0.657	0.039
Parity shifting RT	732.85 (668.52, 810.24)	61.84	763.13 (670.50, 826.75)	68.11	−0.952	0.341	0.083
Parity shifting ACC	93.75 (81.25, 100.00)	63.95	93.75 (87.50, 100.00)	66.04	−0.329	0.743	0.029
Size and parity shifting RT	866.38 (435.33, 1103.73)	56.55	1017.56 (813.24, 1202.61)	73.32	−2.548	0.011	0.222
Size and parity shifting ACC	75.00 (59.38, 87.50)	62.27	78.13 (59.38, 87.69)	67.69	−0.826	0.409	0.072

EG, expert group; NG, novice group; M, mean; SD, standard deviation; RT, response time; ACC, accuracy; Mdn, median; *Q1*, first quartile; *Q3*, third quartile; *ES r*, effect size.

### 3.6 The correlation between ice hockey training experience and executive function task performance

**Figure 1:**
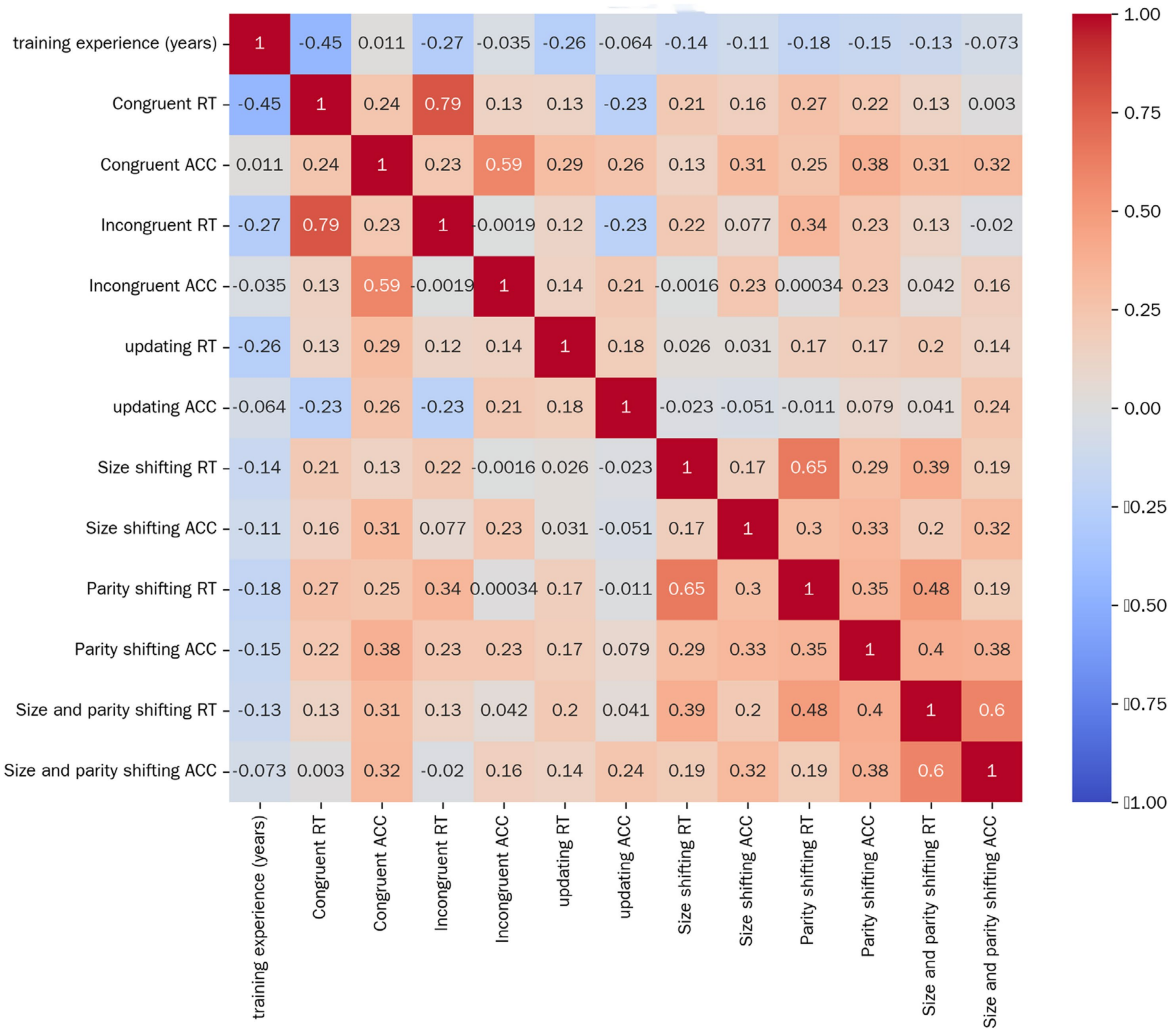
Analysis of the results of the correlation between training experience and executive function task performance among adolescent ice hockey players.

As shown in Figure 1, the data from the correlation analysis between the training experience of adolescent ice hockey players and their performance in executive function tasks reveal that there is a significant negative correlation (*p* < 0.05) between the training duration of this group and the response times of multiple executive function indicators. Specifically, the correlation coefficient is −0.450 in the congruent inhibition condition, −0.267 in the incongruent inhibition condition, −0.257 in the updating task, and −0.185 in the parity shifting task.

## 4 Discussion

This study investigated and compared the performance of expert and novice groups of adolescent ice hockey players on executive function tasks. It was found that the expert group demonstrated superior performance in executive function tasks, particularly in terms of response times for inhibition, updating, and size and parity shifting. Additionally, there was a significant negative correlation between the training years of adolescent ice hockey players and their response times for inhibition, updating, and parity shifting.

### 4.1 The expert group has more remarkable advantages in executive function

The results of this study showed that, compared with the novice group, the expert group of adolescent ice hockey players had shorter response times in the congruent and incongruent conditions of the Flanker task, the response time in the 2-back task, and the response time in the size and parity switching of the More-odd shifting task. Therefore, the expert group demonstrated more excellent performance in executive function tasks. The conclusions of this study are consistent with those of previous research on soccer players (Verburgh et al., 2014; Vestberg et al., 2017), basketball players (López et al., 2017), and ice hockey players (Lundgren et al., 2016; Tamás et al., 2024), all of which have confirmed that expert athletes outperform novice athletes in executive function tasks. The findings of this study indicate that the expert group had longer training years, higher weekly training frequency, and longer single training duration, with richer ice hockey experience. It is plausible that such long-term training has accumulated key cognitive resources that facilitate the development of executive function. Sustained specialized training not only enhances the automation of athletes’ motor skills, but also systematically hones their attention allocation, working memory updating, and response inhibition capabilities through complex tactical decision-making, situational anticipation, and team collaboration tasks, thereby promoting the structural improvement of executive function. Therefore, the superior executive function advantages of adolescent ice hockey players in the expert group can be explained from multiple perspectives, such as the characteristics of sports training, adaptation to cognitive demands, and changes in neural mechanisms.

Firstly, the uniqueness of ice hockey places extremely high demands on executive functions, and long-term ice hockey training can specifically enhance related cognitive abilities. In ice hockey games, athletes need to ignore irrelevant distractions (such as opponents’ feints) during fast-paced offensive and defensive plays and focus on core goals (such as controlling the puck, passing, or defending). This continuous “anti-interference demand” strengthens the brain’s inhibition control ability, enabling the expert group to respond faster in the Flanker task, which requires inhibiting interference from irrelevant information. In ice hockey matches, athletes need to update dynamic information in real time (such as teammates’ positions, opponents’ tactics, and the trajectory of the puck) and quickly replace outdated information (such as previous offensive and defensive strategies). This long-term training improves the “information updating efficiency” of working memory, making the expert group perform better in the 2-back task, which requires continuous updating of memory content. In addition, the transition between offense and defense in ice hockey games is extremely fast, and athletes need to flexibly switch between different task rules (such as puck control strategies and defensive positions) frequently. This high-intensity “task-switching demand” enhances cognitive flexibility, allowing the expert group to respond more quickly in size and parity switching tasks.

Secondly, through years of systematic training, the expert group has developed a highly automated “cognition-action” collaborative pattern (MacIntyre et al., 2014). Due to long-term training, the expert group has formed “automated connections” between rule judgment, information processing, and action execution for relevant tasks (for example, they can respond quickly without deliberate thinking after seeing a signal) (Gao et al., 2025). This saves cognitive resources, thereby shortening response time. In contrast, when novice players perform cognitive tasks, they may need to allocate more cognitive resources to basic links such as “understanding task rules” and “controlling action output”, resulting in longer response times (Gao et al., 2025).

Thirdly, long-term ice hockey training may lead to plastic changes in the structure and function of athletes’ brains (DiFabio et al., 2023). Neuroimaging studies (Hvid et al., 2021; Lin et al., 2012; Patten et al., 2013) have demonstrated that long-term exercise can increase the gray matter density and neuronal connection efficiency of the prefrontal cortex, thereby enhancing the speed of information transmission. The prefrontal cortex is the core brain region responsible for executive function (Friedman and Robbins, 2022). In particular, open sports with rich environmental stimuli, which require real-time response to dynamically changing environments, opponents’ behaviors and unexpected situations, can reshape brain structure and function through continuous sensory input, decision-making training and neural regulation (Shi and Feng, 2022; Takahashi and Grove, 2023; Zhang et al., 2024). In addition, exercise may promote the secretion of neurotransmitters such as dopamine and norepinephrine, which are closely related to attention and reaction speed, further optimizing the performance in executive function tasks (Arnsten and Li, 2005; Bove et al., 1984; Logue and Gould, 2014).

In summary, the superior executive function of the expert group is the combined effect of ice hockey-specific cognitive demands and adaptive neural mechanisms underpinned by extensive sports experience. The continuous requirements of ice hockey on inhibition, updating, and switching functions are transformed into stable improvements in cognitive abilities through training, which are ultimately reflected in shorter response times in laboratory tasks. In contrast, the novice group has relatively limited ice hockey experience and receives lower levels of cognitive stimulation from ice hockey training, and thus may fail to translate these experiences into beneficial effects on executive function. This conclusion also supports, to a certain extent, the finding that there is a significant negative correlation between training duration and response time in executive function tasks.

### 4.2 The expert group did not demonstrate higher accuracy in executive function tasks

The results of this study revealed that expert-group players demonstrated faster reaction speeds in executive function tasks but did not exhibit higher accuracy. This phenomenon may be related to the speed-prioritized cognitive pattern shaped by long-term ice hockey training. Furthermore, this study also found that ice hockey players exhibit a speed-accuracy trade-off, which further supports this finding.

The core competitive demands of ice hockey (such as making rapid decisions in high-speed confrontations and instantaneous switching between offense and defense) determine that training prioritizes the enhancement of “reaction speed.” Players need to judge the trajectory of the puck and opponents’ movements within milliseconds and initiate response strategies; the cost of a “slow reaction” (such as missing an offensive opportunity or being broken through by opponents) is far greater than that of an “occasional mistake.” This long-term training-induced “speed-prioritized” strategy is internalized into a stable cognitive habit. When transferred to laboratory tasks, the brain defaults to taking “shortening response time” as the primary goal, even triggering responses before information is fully processed, thereby sacrificing part of the accuracy.

In addition, the performance of executive functions is significantly “context-dependent,” and there are essential differences between the core demands of laboratory tasks and those of ice hockey arenas. In real arenas, task goals are ambiguous and dynamically changing, allowing for “probabilistic decision-making” (i.e., “fast and likely correct” is better than “slow and absolutely correct”), and players’ cognitive strategies focus more on “efficient adaptation” rather than “absolute precision.” In contrast, laboratory tasks have clear and static rules (such as fixed stimulus presentation and response requirements) and emphasize “strict adherence to a single set of rules.” The level of accuracy in such tasks often depends on “strict compliance with fixed rules.” Therefore, this discrepancy makes it difficult for the “dynamically adaptive cognitive strategies” strengthened by ice hockey training to translate into an advantage in accuracy in laboratory-based “static rule-based tasks.”

Finally, the speed-accuracy trade-off mechanism in cognitive psychology comes into play here. Individuals actively adjust resource allocation according to the priority of task goals. Due to long-term training, the expert group has formed a “speed-prioritized” trade-off pattern. Their brains tend to “output responses quickly” in the early stage of information processing, rather than allocating more resources for “secondary verification.” In contrast, the novice group, lacking training in “speed pressure,” is more likely to default to the strategy of “ensuring accuracy” even if their reactions are slower. This difference in active choice is not a “deficiency in ability” but a “functional adjustment” made by the expert group to adapt to the needs of ice hockey. In real game scenarios, this trade-off can maximize competitive performance; however, in laboratory tasks where “accuracy” is the core indicator, it instead appears as a “disadvantage.”

In summary, the fact that the expert group did not demonstrate higher accuracy essentially results from the interaction between the specific demands of ice hockey training and the task context. This outcome does not negate the improvement of executive functions brought about by ice hockey training; rather, it suggests that the impact of sports on cognition is “selective.” Its advantages are more likely to be reflected in dimensions highly related to the core demands of the sport (such as reaction speed), rather than in all cognitive indicators.

### 4.3 Ice hockey experience is positively correlated with executive function

The results of this study found that there is a significant positive correlation between ice hockey training experience and adolescents’ performance in executive function tasks. The findings of this study are similar to those of previous studies (Heilmann et al., 2023; Marzocchi et al., 2020).

Ice hockey is a high-speed, confrontational team sport. During a game, players need to process multiple pieces of information simultaneously, including maintaining balance while gliding on the ice, tracking changes in the positions of teammates and opponents, following the rapid trajectory of the puck during passes, and observing the referee’s penalty signals. Such an environment requires adolescents to maintain sustained selective attention (filtering out irrelevant information and focusing on key targets) and divided attention (attending to multiple tasks at the same time) during training and competitions. Long-term training strengthens the neural connections in the prefrontal cortex of the brain (the core region responsible for executive functions), enabling adolescents to perform better in executive function tasks (such as attention switching and target tracking tests) (Demirakca et al., 2016; Moore et al., 2022). In addition, the situation in ice hockey games changes rapidly, and players need to make various complex decisions within milliseconds. This high-frequency training in immediate decision-making can improve adolescents’ updating and shifting functions.

Finally, ice hockey training includes a great deal of physical fitness training such as endurance and coordination, and the physical activity level of adolescents is closely related to brain development (Erickson et al., 2015; da Gomes Silva and Arida, 2015). Neuroscientific studies (Hvid et al., 2021; Lin et al., 2012; Patten et al., 2013; Querido and Sheel, 2007) have demonstrated that regular exercise can increase cerebral blood flow and neuroplasticity in the prefrontal cortex, and promote the secretion of neurotransmitters such as dopamine that are crucial for the regulation of executive function. Therefore, ice hockey training not only improves executive functions through cognitive challenges, but also provides a better physiological foundation for neural development by enhancing physical fitness.

In summary, ice hockey training systematically promotes the development of core elements of adolescents’ executive functions through the multi-dimensional effects of “attention training, decision-making exercise, and physical-mental coordination”, thereby forming a significant positive correlation between the two. Essentially, this correlation is the result of the long-term shaping of the brain’s cognitive control system by sports experience.

### 4.4 The value and significance of this study

At the theoretical level, existing research on the executive functions of ice hockey players is relatively scarce, especially lacking empirical evidence from Chinese samples. This study, with Chinese adolescent ice hockey players as participants, systematically adopted standardized computer-based testing tasks such as Flanker, 2-back, and More-odd shifting for the first time, clarified the specific advantages of ice hockey players in the three dimensions of executive functions (inhibition, updating, and shifting), and supplemented localized data for this field. In addition, this study confirmed that there is a significant correlation between long-term ice hockey training and the improvement of executive functions, and this correlation is reflected in the optimization of reaction speed. This result supports the “sports-specific cognitive enhancement” theory, that is, the high confrontation and fast-paced characteristics of ice hockey can specifically strengthen the brain’s advanced cognitive control ability, providing a new perspective for understanding how sports training shape cognitive functions.

At the practical level, this study found that the expert group significantly outperformed the novice group in terms of response time in the inhibition task (Flanker), updating task (2-back), and shifting task (More-odd shifting), with longer training duration associated with faster reaction speeds. This suggests that executive function can serve as a potential indicator for ice hockey player selection, and in particular, cognitive tests related to reaction speed can assist in identifying promising young players. In addition, based on the results of this study, ice hockey training can be specifically integrated with content aimed at improving executive function. For example, inhibition control can be strengthened by simulating distracting scenarios in competitions, working memory updating ability can be enhanced through dynamic information updating training, and cognitive flexibility can be improved through rapid offensive and defensive transition training, thereby enhancing the precision and efficiency of training.

In summary, this study not only enriches the theoretical system in the field of sports cognition but also provides operable scientific basis for talent selection and training practice in ice hockey.

### 4.5 Limitations of this study

Although this study confirms that adolescent ice hockey players in the expert group have superior executive function, there are certain limitations. Firstly, the sample representativeness has obvious limitations. In the sample size calculation, this study adopted the effect size from a meta-analysis (Scharfen and Memmert, 2019). However, this meta-analysis was not a quantitative summary exclusively focusing on executive function, which may have led to calculation errors. In addition, all participants in this study were male athletes from Shenyang, China, with no female subjects included. This was because the study aimed to avoid potential sample interference caused by a small number of female participants, and thus the female group was ultimately excluded. Secondly, there are multiple deficiencies in the research methodology. The cross-sectional design has inherent flaws: it cannot fully distinguish the independent effects of innate traits and acquired training, nor can it establish a causal relationship between them. Furthermore, although the computerized test tasks employed in this study are standardized tools in the field of executive function assessment, there is currently a lack of unified normative data for Chinese adolescent populations. This makes it difficult to directly compare and analyze the test results with those of the general adolescent population. Meanwhile, there are significant discrepancies between the static, single-target task paradigm of these laboratory tests and the dynamic context of actual ice hockey matches. Such a paradigm may not be sufficient to capture the executive function advantages exhibited by athletes in real-game scenarios. Thirdly, the control of confounding factors needs further optimization. This study did not strictly match non-sport-related variables such as age, baseline cognitive level, family socioeconomic status, height, and weight, which constitutes a certain methodological limitation. Additionally, athletes may autonomously choose higher training loads based on their own capabilities, and this study failed to effectively control for this self-selection effect of training load, which may interfere with the accuracy of the research conclusions.

Based on the aforementioned limitations, the following prospects for future research are proposed. Firstly, expand the sample scope to improve sample representativeness. For sample size calculation, obtain targeted effect sizes through pilot experiments, and on this basis, calculate the sample size scientifically to ensure that the sample size is sufficient to support the statistical validity of the research conclusions. In terms of sample representativeness, expand the geographical coverage of the sample, break through the limitation of a single city, and include adolescent ice hockey players from different regions of China to enhance the geographical representativeness of the sample. In addition, eliminate gender restrictions, reasonably include female adolescent ice hockey players, avoid interference caused by minority groups by increasing the sample size of females, and conduct a comparative analysis of the differences and commonalities in executive functions among athletes of different genders. Secondly, optimize the research design and assessment tools to enhance the causal explanatory power and ecological validity of the study. By conducting long-term tracking of the training process and developmental trajectory of executive functions of adolescent athletes, distinguish the independent effects and interaction effects of innate traits and acquired training on executive functions. Furthermore, improve the executive function assessment system to enhance the applicability and ecological validity of the assessment. On the one hand, promote the establishment of unified normative data of computerized executive function tests for Chinese adolescent groups, so as to facilitate horizontal comparison with the general adolescent group and clarify the unique advantages of executive functions in the athlete group. On the other hand, enrich the types of assessment tasks; on the basis of retaining standardized laboratory tests, develop dynamic assessment tasks that are close to the real-game scenarios of ice hockey. Thirdly, improve the control of confounding factors to increase the accuracy of the research conclusions. At the stage of research design, clearly identify non-sport-related variables that may affect executive functions, and adopt methods such as stratified sampling and matched design to ensure the balance of the above variables between the expert group and the novice group. In addition, on the basis of addressing the existing limitations, future research can further deepen the exploration of the internal mechanisms underlying the association between executive functions and sports training.

## 5 Conclusion

This study systematically compared the executive function performance between the expert group (municipal team athletes) and the novice group (club novices) of Chinese youth ice hockey players through Flanker, 2-back, and More-odd shifting tasks. It was found that the expert group had significantly shorter response times than the novice group in the core dimensions of executive function, including inhibition function (both congruent and incongruent conditions of the Flanker task), updating function (2-back task), and shifting function (size-parity shifting condition of the More-odd shifting task). In addition, there was a significant negative correlation between the training years of youth ice hockey players and the response times in executive function tasks, meaning that the longer the training duration, the faster the reaction speed in executive function tasks. In summary, this study confirmed the positive impact of long-term ice hockey training on the executive function of adolescents, and clarified that its advantages are mainly reflected in the dimension of reaction speed. It provides empirical evidence for understanding the relationship between sports training and cognitive development, and also offers a scientific reference for the selection and targeted training of youth ice hockey players.

## Data Availability

The raw data supporting the conclusions of this article will be made available by the authors, without undue reservation.
